# Expression Profile of the Chromosome 14 MicroRNA Cluster (C14MC) Ortholog in Equine Maternal Circulation throughout Pregnancy and Its Potential Implications

**DOI:** 10.3390/ijms20246285

**Published:** 2019-12-13

**Authors:** Pouya Dini, Hossam El-Sheikh Ali, Mariano Carossino, Shavahn C. Loux, A. Esteller-Vico, Kirsten E. Scoggin, Peter Daels, Barry A. Ball

**Affiliations:** 1 Faculty of Veterinary Medicine, Ghent University, 9820 Merelbeke, Belgium; pouya.dini@uky.edu (P.D.); Peter.Daels@UGent.be (P.D.); 2 Gluck Equine Research Center, Department of Veterinary Science, University of Kentucky, Lexington, KY 40503, USA; hossam.elsheikh@uky.edu (H.E.-S.A.); mcarossino1@lsu.edu (M.C.); shavahn.loux@uky.edu (S.C.L.); kirsten.scoggin@uky.edu (K.E.S.); 3 Theriogenology Department, Faculty of Veterinary Medicine, University of Mansoura, Mansoura 35516, Egypt; 4 Louisiana Animal Disease Diagnostic Laboratory and Department of Pathobiological Sciences, School of Veterinary Medicine, Louisiana State University, Baton Rouge, LA 70803, USA; 5 Department of Biomedical and Diagnostic Sciences, College of Veterinary Medicine, University of Tennessee, Knoxville, TN 37996, USA; aestellervico@uky.edu

**Keywords:** C14MC, C24MC, equine chromosome 24, pregnancy, circulating microRNAs, biomarker, mare, embryo

## Abstract

Equine chromosome 24 microRNA cluster (C24MC), the ortholog of human C14MC, is a pregnancy-related miRNA cluster. This cluster is believed to be implicated in embryonic, fetal, and placental development. The current study aimed to characterize the expression profile of this cluster in maternal circulation throughout equine gestation. The expression profile of miRNAs belonging to this cluster was analyzed in the serum of non-pregnant (diestrus), pregnant (25 d, 45 d, 4 mo, 6 mo, 10 mo), and postpartum mares. Among the miRNAs examined, 11 miRNAs were differentially expressed across the analyzed time-points. Four of these miRNAs (*eca-miR-1247-3p*, *eca-miR-134-5p*, *eca-miR-382-5p,* and *eca-miR-433-3p*) were found to be enriched in the serum of pregnant mares at Day 25 relative to non-pregnant mares. To further assess the accuracy of these miRNAs in differentiating pregnant (25 d) from non-pregnant mares, receiver operating characteristic (ROC) analysis was performed for each of these miRNAs, revealing that *eca-miR-1247-3p* and *eca-miR-134-5p* had the highest accuracy (AUC_ROC_ = 0.92 and 0.91, respectively; *p* < 0.05). Moreover, *eca-miR-1247-3p*, *eca-miR-134-5p*, *eca-miR-409-3p,* and *eca-miR-379-5p* were enriched in the serum of Day 45 pregnant mares. Among those miRNAs, *eca-miR-1247-3p* and *eca-miR-409-3p* retained the highest accuracy as shown by ROC analysis. GO analysis revealed that these miRNAs are mainly implicated in nervous system development as well as organ development. Using in situ hybridization, we localized *eca-miR-409-3p* in the developing embryo (25 d) and extra-embryonic membranes (25 and 45 d). In conclusion, the present study is the first to elucidate the circulating maternal profile of C24MC-associated miRNAs throughout pregnancy and to suggest that serum *eca-miR-1247-3p*, *eca-miR-134-5p*, and *eca-miR-409-3p* could be used as pregnancy-specific markers during early gestation (25 and 45 d). Overall, the high abundance of these embryo-derived miRNAs in the maternal circulation suggests an embryo-maternal communication during the equine early pregnancy.

## 1. Introduction

MicroRNAs (miRNAs) are a class of small non-coding RNA that post-transcriptionally regulate protein-coding mRNAs [1]. MiRNAs are expressed in a variety of cell types as biological regulators. They play a fundamental role in the regulation of a variety of developmental and physiological processes, such as cell proliferation, growth, metabolism, communication, apoptosis, and death [2,3,4]. Aberrant expression of miRNAs has been associated with many pathological disorders and diseases, including cancer and abnormalities during pregnancy [5,6,7,8]. Occasionally throughout the genome, a group of two or more miRNAs are transcribed from physically adjacent miRNA genes (within 10 kb), which form a cluster [9,10]. These miRNA clusters have been found to be exclusively or preferentially expressed in a tissue-specific manner [11]. This tissue expression is at least partly reflected in the circulation (i.e., circulating miRNAs) and could be detected in plasma and serum with much greater sensitivity than protein markers [12,13]. The measurability, sensitivity, and stability (approximately five day half-life) of circulating miRNAs [13,14] empowered them to emerge as biomarkers for several physiological and pathological conditions, including diabetes [15], cancer [16,17], cardiovascular diseases [18,19], and skeletal diseases [20].

In reproductive biology, miRNAs are involved in folliculogenesis, corpus luteum formation, endometrial functions, embryogenesis, maternal recognition of pregnancy, embryo implantation, as well as placental development [21,22,23,24,25,26,27,28,29]. Recently, there has been growing interest in pregnancy-related and/or placenta-specific miRNAs in mammals [7,21,30,31,32,33]. This interest led to the identification of several circulating miRNAs as biomarkers for early pregnancy diagnosis [34], prediction and/or diagnosis of embryonic loss [35,36], ectopic pregnancy [37,38], pre-eclampsia [39,40], intrauterine growth restriction [40,41], placental infection [41], and preterm labor [41,42]. In humans, the chromosome 14 miRNA cluster (C14MC) is one of the largest pregnancy-related miRNA clusters and consists of 52 miRNAs [21]. In the horse, C24MC is the orthologous cluster to human C14MC [29]. Recently, our group has elucidated the kinetics of C24MC in the chorioallantoic membrane (CAM) throughout equine gestation [29]. We demonstrated that C24MC-associated miRNAs were upregulated in CAM during the early stages of equine pregnancy, followed by a downregulation later in gestation [29]. Moreover, functional analysis of mRNAs targeted by this miRNAs cluster suggested that C24MC are involved in embryonic development, endothelial cell migration, and angiogenesis during placental development in the horse [29]. So far, the expression profile of C14MC in maternal circulation throughout gestation has not been elucidated.

We hypothesized that equine C24MC-associated miRNAs, the ortholog of human C14MC, will have a differential expression pattern in maternal circulation throughout gestation. Moreover, identifying the normal profile of these miRNAs in maternal circulation in comparison to non-pregnant mares and postpartum mares could positively impact the development of novel and non-invasive biomarkers for pregnancy diagnosis and prediction of pregnancy outcomes and/or complications. Therefore, this study was designed to evaluate the expression profile of equine C24MC-associated miRNAs in the serum of non-pregnant (diestrus), pregnant (25 days, 45 days, 4 months, 6 months, 10 months) and postpartum mares.

## 2. Results

### 2.1. Equine C24MC Expression in Serum

**Figure 1:**
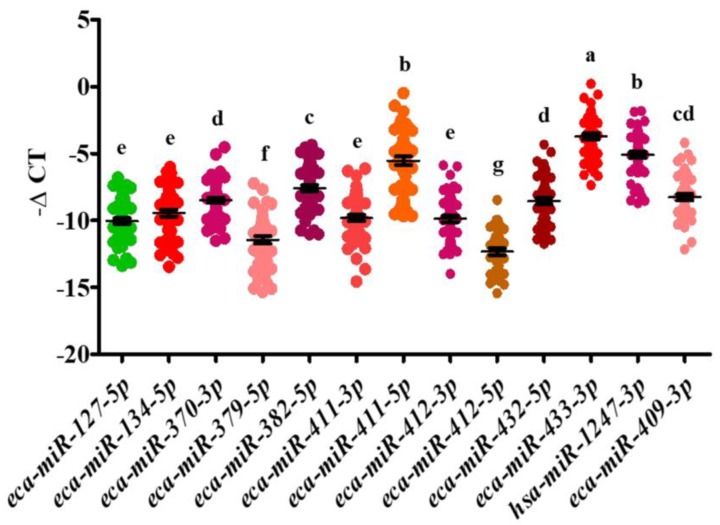
Abundance of the tested miRNAs in equine circulation. Expression of circulating miRNAs was analyzed using RT-qPCR through gestation (25 d, 45 d, 4 mo, 6 mo, and 10 mo), diestrus (non-pregnant) and post-partum period. Expression of each microRNA was normalized to the geometric mean of *eca-miR-10a*, *eca-miR-21,* and *eca-Let-7a*, expressed as -ΔCT. Data are presented as a dot plot and the middle horizontal line represents the mean while error bars represent the standard error of the mean (SEM). Significantly, different samples are indicated by varying superscripts.

**Figure 2:**
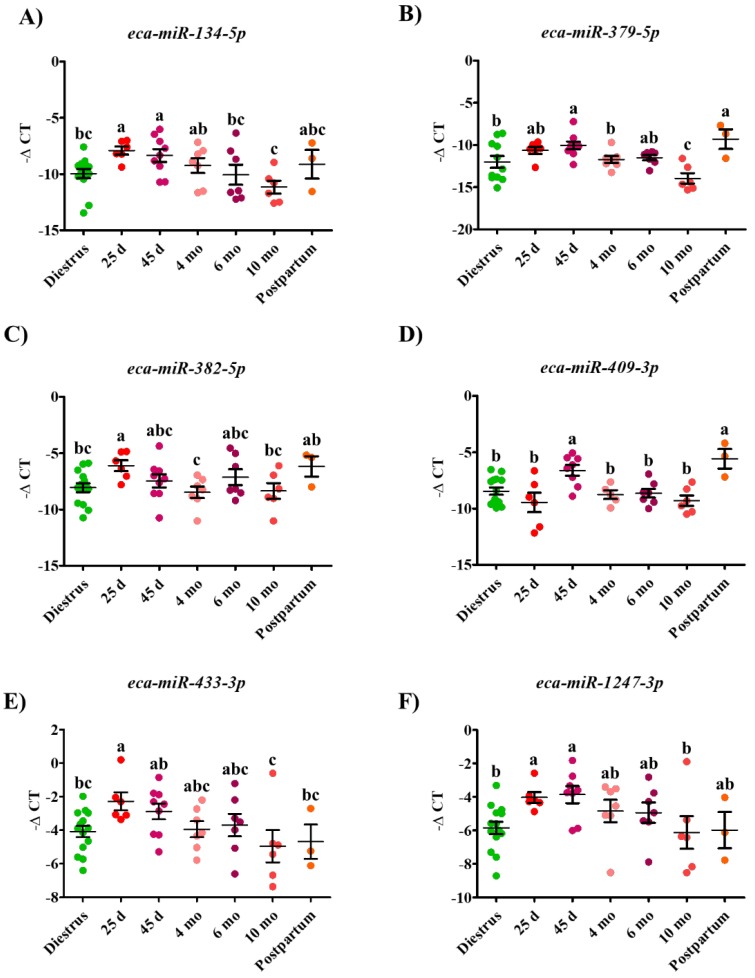
Differential expression profile of *eca-miR-134-5p* (**A**), *eca-miR-379-5p* (**B**), *eca-miR-382-5p* (**C**), *eca-mir-409-3p* (**D**)*, eca-miR-433-3p* (**E**) and *eca-miR-1247-3p* (**F**) during diestrus, gestation (25 d, 45 d, 4 mo, 6 mo and 10 mo) and postpartum period in mares’ circulation. Expression of each microRNA was normalized to the geometric mean of *eca-miR-10a*, *eca-miR-21, and eca-Let-7a*, expressed as -ΔCT. Data are presented as a dot plot and the middle horizontal line represents the mean while error bars represent the standard error of the mean (SEM). Significantly different time-points are indicated by varying superscripts.

**Figure 3:**
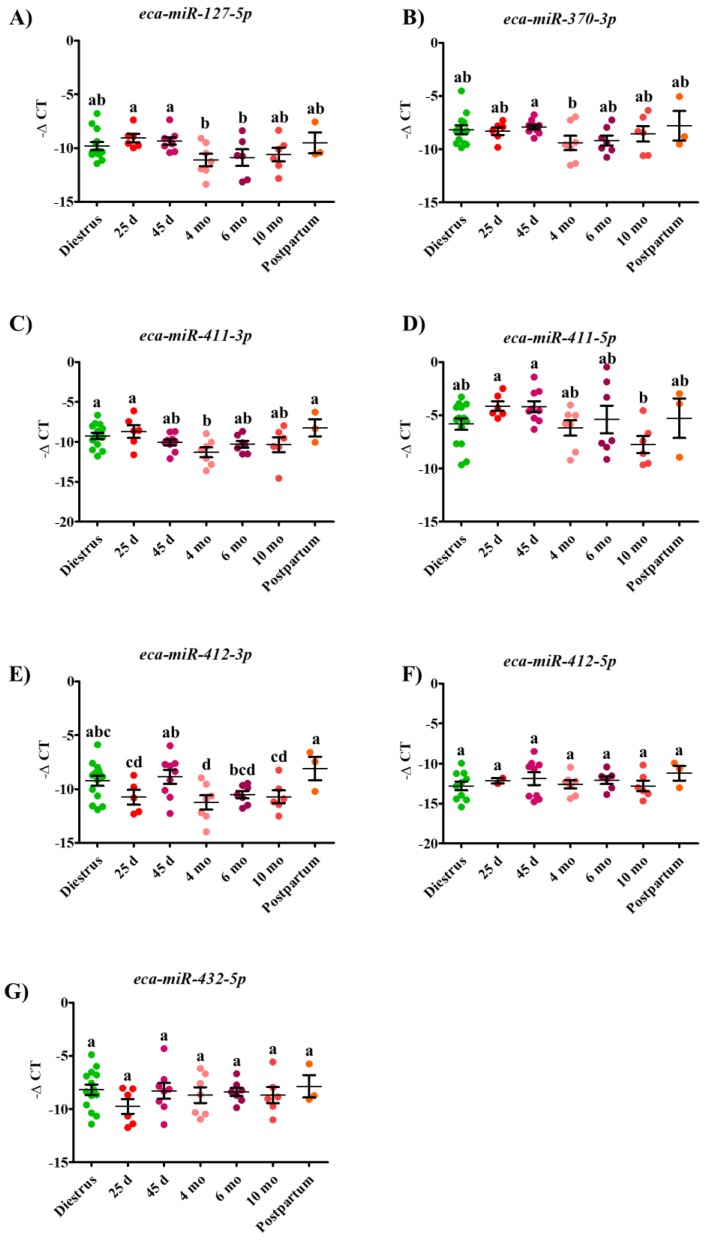
Differential expression profile of *eca-miR-127-3p* (**A**)*, eca-miR-370-3p* (**B**), *eca-miR-411-3p* (**C**), *eca-miR-411-5p* (**D**), *eca-miR-412-3p* (**E**), *eca-miR-412-5p* (**F**) and *eca-miR-432-5p* (**G**) during diestrus, gestation (25 d, 45 d, 4 mo, 6 mo, and 10 mo) and postpartum period in mares circulation. Expression of each microRNA was analyzed using RT-qPCR and normalized to the geometric mean of *eca-miR-10a*, *eca-miR-21,* and *eca-Let-7a*, expressed as -ΔCT. Data are presented as a dot plot and the middle horizontal line represents the mean while error bars represent the standard error of the mean (SEM). Significantly different time-points are indicated by varying superscripts.

To determine the expression pattern of C24MC-associated miRNAs in maternal serum, the expression of candidate miRNAs was evaluated in samples from pregnant (25 d, 45 d, 4 mo, 6 mo, and 10 mo), postpartum and non-pregnant (diestrous) mares. Out of the 26 tested miRNAs, 13 of them showed a persistent expression at all time-points (expressed in all the samples from at least one time points). The remaining 13 miRNAs were not detected in all the samples therefore were excluded from the study. Regarding the abundance of tested miRNAs in equine circulation, we found *eca-miR-433-3p*, *eca-miR-1247-3p*, *eca-miR-134-5p,* and *eca-miR-411-5p* with the greatest abundance in serum (*p <* 0.05). On the other hand, *eca-miR-412-5p* and *eca-miR-379-5p* showed the lowest abundance (*p <* 0.05) (Figure 1). Out of the tested miRNAs, 11 miRNAs were differentially expressed across the analyzed time-points (*p* < 0.05), whereas the expression profile of *eca-miR-412-5p* and *eca-miR-432-5p* did not show any significant fluctuation. The differential expression profile of tested miRNAs is depicted in Figure 2 and Figure 3. The correlation between tested miRNAs expression profiles is illustrated in Table 1.

**Figure 4:**
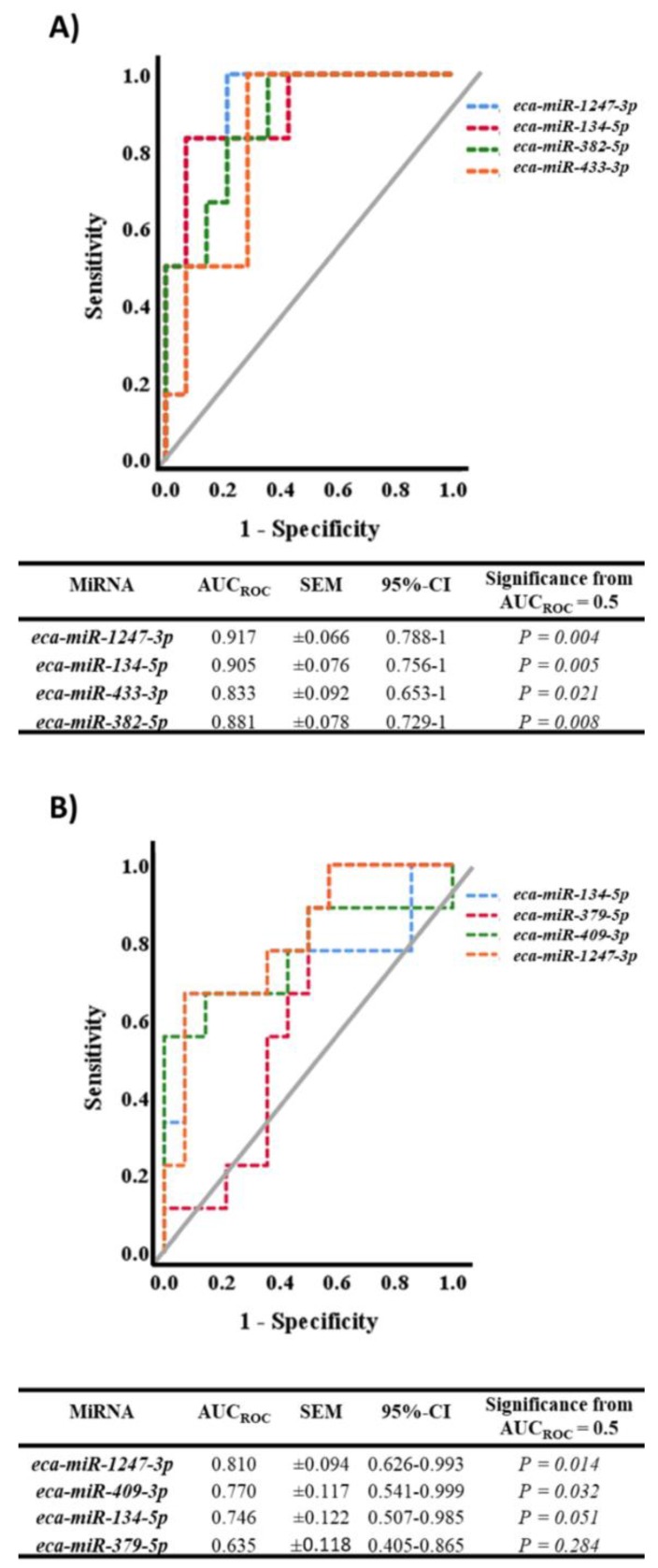
(**A**) Receiver operating characteristic (ROC) curves of circulating *eca-miR-1247-3p*, *eca-miR-134-5p*, *eca-miR-433-3p,* and *eca-miR-382-5p* expression for the discrimination of early pregnant mares (25 d, n = 6) from non-pregnant mares (diestrus, n = 14). (**B**) ROC curves of circulating *eca-miR-1247-3p*, *eca-miR-134-5p*, *eca-mir-409-3p,* and *eca-miR-379-5p* expression for the discrimination of early pregnant mares (45 d, n = 8 for *eca-miR-1247-3p* and *eca-mir-409-3p*, and n = 9 for *eca-miR-134-5p* and *eca-miR-379-5p*) from non-pregnant mares (diestrus, n = 14). Table in subfigure **A** and **B** elucidate the results generated from ROC curve analysis for 25 and 45 d pregnancy dataset, respectively.

Among the differentially expressed miRNAs, four miRNAs (*eca-miR-1247-3p*, *eca-miR-134-5p*, *eca-miR-382-5p,* and *eca-miR-433-3p*) were found to be enriched (*p* < 0.05) in the serum of Day 25 pregnant mares in comparison to non-pregnant (diestrous) mares, as illustrated in Figure 2. Another common feature among these miRNAs is the downregulation (*p* < 0.05) of all of them at 10 mo of gestation in comparison to 25 d of gestation (Figure 2). Additionally, the expression profile of all the expressed miRNAs was positively correlated to each other, with the highest correlation observed between *eca-miR-1247-3p* and *eca-miR-433-3p* (r = 0.92, *p* < 0.01; Table 1). To determine the accuracy of these miRNAs in distinguishing pregnant (25 d) from non-pregnant mares, ROC analysis was performed and demonstrated that *eca-miR-1247-3p* and *eca-miR-134-5p* showed the highest accuracy (AUC_ROC_ = 0.92 and 0.91, respectively; *p < 0.01*) compared to *eca-miR-382-5p* and *eca-miR-433-3p*, which were moderately accurate (AUC_ROC_ = 0.88 and 0.83, respectively; *p* < 0.05) (Figure 4A). Out of these four miRNAs, *eca-miR-1247-3p* and *eca-miR-134-5p* remained enriched (*p* < 0.05) in serum of Day 45 pregnant mares in comparison to non-pregnant mares. Similarly, *eca-miR-409-3p* and *eca-miR-379-5p* expression profiles were enriched in serum of Day 45 pregnant mares in comparison to non-pregnant mares (*p* < 0.05). Again, ROC analysis was performed to determine the accuracy of these miRNAs in differentiating pregnant (45 d) from non-pregnant mares, and indicated that *eca-miR-1247-3p* and *eca-miR-409-3p* have a significant moderate accuracy (AUC_ROC_ = 0.81 and 0.77, respectively; *p* < 0.05) relative to a non-significant AUC_ROC_ for *eca-miR-134-5p* (*p* = 0.05) and *eca-miR-379-5p* (*p* = 0.284), as shown in Figure 4B.

Within the remaining differentially expressed miRNAs, expression of *eca-miR-127-5p* and *eca-miR-411-3p* was significantly higher in serum at 25 d compared to 4 mo, as illustrated in Figure 3. Moreover, *eca-miR-127-5p*, *eca-miR-370-3p*, and *eca-miR-412-3p* were significantly upregulated in serum at 45 d GA compared to 4 mo GA (Figure 3).

### 2.2. Gene Ontology Analysis for the Differnetially Expressed miRNAs

**Figure 5:**
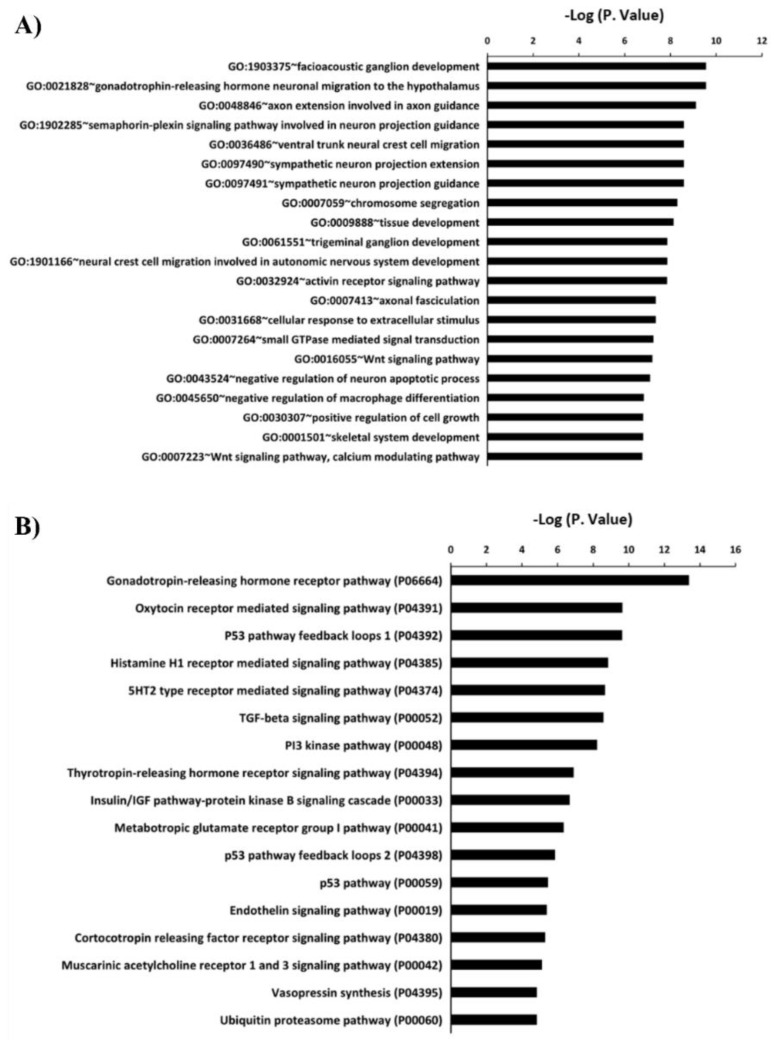
Gene ontology and pathway analysis of C24MC-associated miRNAs. The target mRNAs for *eca-miR-1247-3p*, *eca-miR-134-5p*, *eca-miR-382-5p*, *eca-miR-433-3p*, *eca-miR-409-3p,* and *eca-miR-379-5p* were predicted using IPA and miRDB. (**A**) Functional annotation analysis (Biological process) of the 428 predicted mRNA’ targets using DAVID. (**B**) Pathways predicted for mRNA’s targets using PANTHER.

Computational target prediction for miRNAs was performed to identify putative mRNA targets. A total of 428 target mRNAs were predicted using mirDB.org and IPA for the differentially expressed miRNAs (*eca-miR-134-5p*, *eca-miR-1247-3p*, *eca-miR-382-5p*, *eca-miR-409-3p*, *eca-miR-433-3p*, and *eca-miR-379-5p*). GO enrichment (biological process) analysis and pathway analysis were carried out. GO analysis revealed that these miRNAs are mainly implicated in nervous system development (Figure 5A). Pathway analysis of the same set of miRNAs revealed that they are associated with the Wnt signaling pathway and oxytocin receptor mediated signaling pathway, among others (Figure 5B).

### 2.3. Localization of a Member of Equine C24MC in Equine Extra-Embryonic Membranes and 25 d Embryos

**Figure 6:**
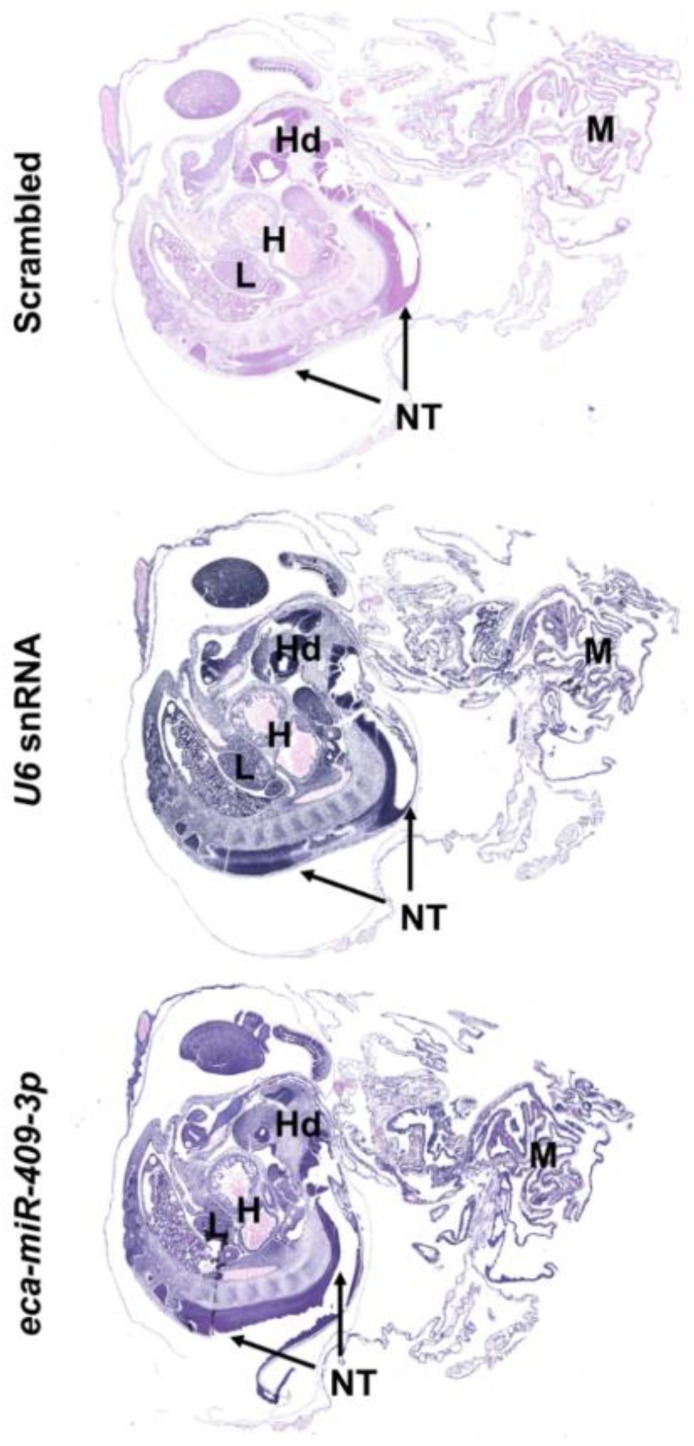
Localization of *eca-miR-409*-3p in the 25 d embryo and extra-embryonic membranes (25 d) by in situ hybridization (bottom image). Extra-embryonic membranes show strong, diffuse signal. Numerous embryonic tissues from the three germ layers including the developing central nervous system (neural tube) show strong signal for *eca-miR-409-3p*. Scrambled and *U6* snRNA were used as negative and positive controls. M, extra-embryonic membranes; *, head; H, heart; NT, neural tube; L, liver. NBT/BCIP (blue) was used as the substrate. Magnification: 25×.

**Figure 7:**
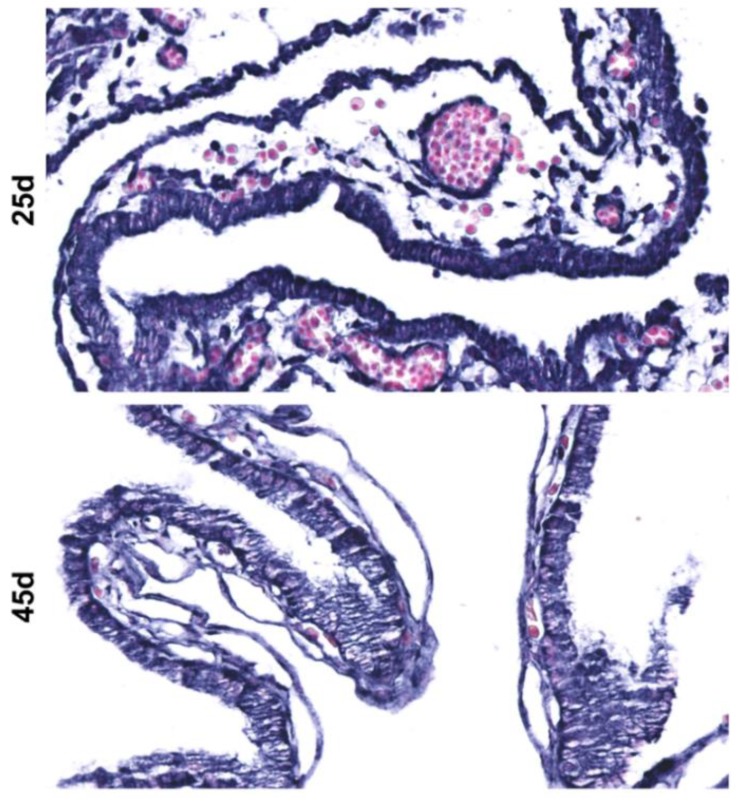
Localization of *eca-miR-409*-3p in the 25 and 45 d extra-embryonic membranes by in situ hybridization. Extra-embryonic membranes show strong cytoplasmic signal for *eca-miR-409-3p*, including trophoblastic cells. NBT/BCIP (blue) was used as the substrate. Magnification: 400×.

In order to study the cellular and subcellular localization of *eca-miR-409-3p* (a member of the C24MC), we performed miRNA in situ hybridization (ISH) on 25 d embryos along with the extra-embryonic membranes of the 25 and 45 d GA. Since *eca-miR-409-3p* had a differential expression pattern during pregnancy and presented a 100% homology to *hsa-miR-409-3p*, this miRNA was chosen as the best candidate for ISH analysis. At 25 d, *eca-miR-409-3p* was intensely expressed in extra-embryonic membranes (Figure 6). In 25 d embryos, the expression of this miRNA was extensive in several developing tissues from the three germ layers (Figure 6). Moreover, extra-embryonic membranes collected at 25 and 45 d shows strong cytoplasmic signal for *eca-miR-409-3p* specifically in trophoblastic cells (Figure 7).

## 3. Discussion

To the best of our knowledge, this is the first study elucidating the expression profile of equine C24MC-associated miRNAs in peripheral circulation of non-pregnant (diestrus), pregnant (25 d, 45 d, 4 mo, 6m, and 10 mo), and postpartum mares. In the present study, four equine miRNAs (*eca-miR-1247-3p*, *eca-miR-134-5p*, *eca-miR-382-5p,* and *eca-miR-433-3p*) were found to be enriched in the serum of Day 25 pregnant mares in comparison to non-pregnant mares. In support of our findings, it has been reported that serum *miR-433-3p* expression is upregulated in Day 19 and 24 pregnant cows [34] and during the first trimester of pregnancy in women [43]. Moreover, it has been reported that serum *hsa-miR-1247-3p* expression is reduced in women with ectopic pregnancy [37] and preeclampsia [39] in comparison to normal pregnant women. To further assess the accuracy of *eca-miR-1247-3p*, *eca-miR-134-5p*, *eca-miR-382-5p,* and *eca-miR-433-3p* in differentiating pregnant (25 d) from non-pregnant mares, ROC analysis was performed and indicated that *eca-miR-1247-3p* followed by *eca-miR-134-5p* retain the highest accuracy. It is worth noting that *miR-134-5p* is highly expressed during embryonic development and becomes restricted to the brain postnatally [44,45]. Moreover, it has been reported that *miR-134-5p* is uniquely correlated in maternal and fetal circulation in women [46]. These facts might suggest that the serum enrichment with *eca-miR-134-5p* during Days 25 and 45 of equine pregnancy is of embryonic origin. Altogether, our findings suggest that *eca-miR-1247-3p* and *eca-miR-134-5p* could be potential biomarkers for pregnancy establishment and/or early embryonic loss at 25 days of equine pregnancy. In equine, it has been suggested that changes in circulating miRNAs could be detected as early as Days 9–13 of pregnancy [47]. However, *eca-miR-1247-3p* and *eca-miR-134-5p* profiles were not investigated in the previously mentioned study. Therefore, further studies are needed to elucidate the expression profile of *eca-miR-1247-3p* and *eca-miR-134-5p* during earlier stages of equine pregnancy.

In the current study, circulating *eca-miR-1247-3p*, *eca-miR-134-5p*, *eca-miR-409-3p,* and *eca-miR-379-5p* expression profile were enriched in 45 d pregnant mares in comparison to non-pregnant. Moreover, ROC analysis indicated that *eca-miR-1247-3p* and *eca-miR-409-3p* have retained the highest accuracy in distinguishing 45 d pregnant mares from non-pregnant mares. Again, these findings suggest that *eca-miR-1247-3p* and *eca-miR-409-3p* could be potential biomarkers for pregnancy establishment and/or early pregnancy loss at Day 45 of equine pregnancy.

The serum enrichment with *eca-miR-1247-3p*, *eca-miR-134-5p*, *eca-miR-382-5p,* and *eca-miR-433-3p* at 25 d pregnancy as well as *eca-miR-1247-3p*, *eca-miR-134-5p*, *eca-miR-409-3p,* and *eca-miR-379-5p* at 45 d pregnancy suggest that these miRNAs are involved in early pregnancy events. In the same line, we found a similar pattern in the expression profile of this cluster of miRNAs in equine chorioallantoic membranes [29]. Moreover, a high expression of C24MC at 45 d was followed by a gradual decline toward the end of the gestation. To gain further insight into the biology of these miRNAs’ targets, GO enrichment (biological process) analysis and pathway analysis were carried out. GO analysis revealed that these miRNAs are mainly implicated in nervous system development as well as organ development, cell-cell signaling, and Wnt signaling pathway, among others. In support of our outcomes, it has been reported that the expression of both *miR-1247-3p* and *miR-409-3p* is significantly downregulated in embryonic neuroblastoma [48,49]. In line with this, ISH analysis of *eca-miR-409-3p* revealed extensive expression in various tissues of the developing embryo including the central nervous system, among others. Moreover, pathway analysis of the same set of miRNAs revealed that they are associated with Wnt signaling pathway and oxytocin receptor mediated signaling pathway, among others. Interestingly, it has been reported that oxytocin responsiveness is altered during early equine pregnancy, and reduced expression of the oxytocin receptor (OXTR) seems to be regulated at the posttranscriptional level rather than the transcriptional level [50]. Our prediction suggests that these miRNAs are potentially involved in oxytocin receptor mediated signaling pathway during early pregnancy in the mare.

In conclusion, the present study is the first to elucidate the maternal circulation profile of C24MC-associated miRNAs throughout equine pregnancy in comparison to non-pregnant (diestrus) and postpartum mares. Moreover, this study introduced serum *eca-miR-1247-3p, eca-miR-134-5p,* and *eca-miR409-3p* as possible non-invasive biomarkers for early pregnancy (25 and 45 d) in mare. We further localized one of these miRNAs (*eca-miR-409-3p*) in the equine 25 d embryo, demonstrating the expression of it in the central nervous system and developing organs. Overall, the high abundance of these embryo-derived miRNAs in the maternal circulation suggests an embryo-maternal communication during the equine early pregnancy.

## 4. Material and Methods

### 4.1. Animal Use

All animal procedures were prospectively approved by and completed in accordance with the Institutional Animal Care and Use Committee of the University of Kentucky (Approval No. #2014-1341, Date: 15 January 2015). All horses used in this study were mixed-breed, ranging between 250 and 550 kg. Mares were housed on pasture with ad libitum grass hay. Gestational age (GA) was determined based on the day of ovulation (Day 0).

### 4.2. Serum Collection and Preparation

For this cross-sectional study, blood sample from pregnant mares at 25 days (25 d; *n* = 5), 45 d (*n* = 9), 4 months (4 mo; *n* = 7), 6 mo (*n* = 9), and 10 mo (*n* = 6) of gestation was collected via jugular venipuncture. Similarly, blood samples were collected from diestrus (Day 7–9 post ovulation, *n* = 14) and postpartum (immediately after normal parturition, *n* = 3). After clotting, the samples were centrifuged at 500× *g* for 10 min at 4 °C. Serum was subsequently removed and stored at −20 °C.

### 4.3. MiRNA Extraction, cDNA Synthesis, and RT-qPCR

MiRNAs were extracted from serum samples (400 μL) using the miRNeasy Micro Kit (Qiagen, Hilden, Germany) according to the manufacturer’s instructions with the following modification: TRIzol™ LS (Life technologies, Carlsbad, CA, USA) was used as the lysis reagent; 200 μL of chloroform was used instead of 140 μL; and the final elution step was performed using 20 μL of RNase-free water instead of 14 μL. Complementary DNA synthesis was carried out using the miScript II RT Kit (Qiagen). Three μL of cDNA product from each sample were combined to make the pooled serum samples. Pooled samples were only used as RT-qPCR inter-plate controls. The expression of mature miRNAs was determined by RT-qPCR using the miScript SYBR Green PCR kits (Qiagen, Hilden, Germany) with the miScript Universal Primer along with miRNA-specific primers according to the manufacturer’s guidelines. Primers were designed using miRprimer software (version 2.0) for the candidate miRNAs [51].

The expression of the C24MC-associated miRNAs (n = 26), which were previously tested in the equine CAM throughout gestation [29], was evaluated in the collected serum samples. The list of miRNA candidates and primer sequences is provided in Table 2. The primers` efficiency was verified on the pooled samples. Primers with CT values <35 that did not produce primer-dimers were used for further experimentation. Otherwise, primers were re-designed and re-tested. The primers that did not yield amplified PCR products in all the samples from at least one of the time-points were excluded from further analysis. *Eca-miR-10a* (gcagtaccctgtagatccga), *eca-miR-21* (gcagtagcttatcagactgatg), and *eca-Let-7a* (gcagtgaggtagtaggttg) were used as reference genes for serum [52]. A DNA melting curve was generated to discriminate between specific and non-specific amplification products. Real-Time qPCR was performed in triplicate for all samples [53,54]. PCR efficiencies were calculated using LinRegPCR (version 2012.0; http://www.hartfaalcentrum.nl) to ensure that all primers resulted in PCR efficiencies between 1.8 and 2.1.

### 4.4. Target Prediction for the miRNA Which Were Highly Expressed during Early Pregnancy (25 and 45 d)

Predicted targets for the six highly expressed miRNAs at 25 and 45 d were selected from IPA (only the Experimentally Observed or High Predicted) and miRDB.org (Target Score >80). Next, to predict functions of C24MC-associated miRNAs, the biological functions and physiological pathways of the target mRNA were analyzed by DAVID and Protein ANalysis THrough Evolutionary Relationships Classification System (PANTHER; Release 13.1) ontology classification system, respectively [55,56].

### 4.5. Eca-miR-409-3p Localization by In Situ Hybridization

The embryo (25 d) and extra-embryonic membranes (25 and 45 d) were retrieved by uterine lavage [29]. The tissue samples were fixed in 10% formaldehyde for 24 h, transferred to 70% methanol and paraffin embedded [29]. The expression of *eca-miR-409-3p* was investigated by chromogenic ISH using a dual digoxigenin (DIG)-labeled LNA™ probe specific to hsa-miR-409-3p (610701-360; miRCURY LNA™, Exiqon, Vedbaek, Denmark) as previously described [29] on 25 d embryos (n = 3) along with the extra-embryonic membranes. A dual DIG-labeled LNA™ probe specific to U6 snRNA (#699002-360; Exiqon) and a scrambled miRNA probe (#699003-360; Exiqon) were used as positive and negative controls, respectively.

### 4.6. Data Analysis

Delta CT (ΔCT) values for serum samples were calculated where ΔCT = (the CT values of the miRNA of interest—the CT values of all three reference miRNAs [geometric mean]) [57,58]. Results are presented as −ΔCT (negative ΔCT is more intuitive than ΔCT). The expressions of C24MC-associated miRNAs were compared across GA, postpartum and with samples from non-pregnant mares using one-way analysis of variance (one-way ANOVA) followed by a pairwise comparison of means using T-test. Significance was set at *p* < 0.05. Pearson’s correlation was performed to analyze the relationship between the expression profile of all the tested miRNAs. Receiver operating characteristic (ROC) curve analysis was carried out to assess the accuracy of *eca-miR-1247-3p*, *eca-miR-134-5p*, *eca-miR-382-5p,* and *eca-miR-433-3p* (differentially expressed miRNAs at 25 d) in differentiating pregnant (25 d) from non-pregnant (diestrus) mares. Moreover, ROC curve analysis was carried out to assess the accuracy of *eca-miR-1247-3p*, *eca-miR-134-5p*, *eca-miR-409-3p*, and *eca-miR-379-5p* (differentially expressed miRNAs at 45 d) in distinguishing pregnant (45 d) from non-pregnant (diestrus) mares. The area under the ROC curve (AUC_ROC_) was estimated and used to evaluate the accuracy of the tested miRNAs [59,60]. In brief, the AUC_ROC_ was inferred as noninformative (AUC_ROC_ ≤ 0.5), poorly accurate (0.5 < AUC_ROC_ ≤ 0.7), moderately accurate (0.7 < AUC_ROC_ ≤ 0.9), highly accurate (0.9 < AUC_ROC_ < 1), or perfect (AUC_ROC_ = 1) [61]. The AUC_ROC_ for each parameter was compared with the expected value (AUC_ROC_ = 0.5) under the null hypothesis of a noninformative test.

## Figures and Tables

**Table 1:** The correlation between tested miRNAs expression profiles.

	*eca-miR-134-5p*	*eca-miR-370-3p*	*eca-miR-379-5p*	*eca-miR-382-5p*	*eca-miR-411-3p*	*eca-miR-411-5p*	*eca-miR-412-3p*	*eca-miR-412-5p*	*eca-miR-432-5p*	*eca-miR-433-3p*	*hsa-miR-1247-3p*	*eca-miR-409-3p*
*eca-miR-127-5p*	0.448 **	0.713 **	0.307 *	0.348 *	0.527 **	0.428 **	0.493 **	0.101	0.163	0.405 **	0.310 *	0.101
*eca-miR-134-5p*	1	0.236	0.482 **	0.520 **	0.255	0.882 **	0.326 *	0.079	0.23	0.597 **	0.614 **	0.313 *
*eca-miR-370-3p*		1	0.236	0.261	0.499 **	0.241	0.512 **	0.207	0.282 *	0.296 *	0.208	0.21
*eca-miR-379-5p*			1	0.560 **	0.259	0.562 **	0.511 **	0.251	0.242	0.253	0.303 *	0.510 **
*eca-miR-382-5p*				1	0.498 **	0.641 **	0.393 **	0.053	0.295*	0.385 **	0.357 **	0.211
*eca-miR-411-3p*					1	0.314 *	0.457 **	0.074	0.122	0.289 *	0.162	0.157
*eca-miR-411-5p*						1	0.362 **	0.058	0.326 *	0.634 **	0.636 **	0.246
*eca-miR-412-3p*							1	0.287	0.187	0.073	0.063	0.468 **
*eca-miR-412-5p*								1	−0.012	0.208	0.239	0.135
*eca-miR-432-5p*									1	0.236	0.316 *	0.461 **
*eca-miR-433-3p*										1	0.919 **	0.053
*esa-miR-1247-3p*											1	0.108

* *p* < 0.05; ** *p* < 0.01.

**Table 2:** List of selected C24MC-associated miRNAs evaluated by RT-qPCR and their respective primer sequences.

MiRNA ID	Accession ID *	Mature Sequence	Forward Primer
*eca-miR-127-5p*	MIMAT0004604	cugaagcucagagggcucugau	ctgaagctcagagggct
*eca-miR-134-5p*	MIMAT0013127	ugugacugguugaccagagggg	gcagtgtgactggttgac
*eca-miR-323-3p*	MIMAT0013132	cacauuacacggucgaccucu	gcagcacattacacggt
*eca-miR-323-5p*	MIMAT0013131	aggugguccguggcgcguucgc	ccgtggcgcgtt
*eca-miR-369-3p*	MIMAT0013141	aauaauacaugguugaucuuu	cagcgcagaataatacatggt
*eca-miR-370-3p*	MIMAT0013142	gccugcugggguggaaccuggu	cctgctggggtgga
*eca-miR-370-5p*	MIMAT0026483	caggucacgucucugcaguuac	cagcaggtcacgtctct
*eca-miR-379-5p*	MIMAT0013147	ugguagacuauggaacguagg	cagtggtagactatggaacg
*eca-miR-382-5p*	MIMAT0013150	gaaguuguucgugguggauucg	aggaagttgttcgtggtg
*eca-miR-3958-3p*	MIMAT0034486	cagauauugcacgguugaucucuu	gcagatattgcacggttga
*eca-miR-3958-5p*	MIMAT0019275	agguuguccgugauguauuugc	agaggttgtccgtgatgt
*eca-miR-409-3p*	MIMAT0013152	gaauguugcucggugaaccccu	aggaatgttgctcggtga
*eca-miR-411-3p*	MIMAT0013154	uauguaacacgguccacuaacc	cagtatgtaacacggtccac
*eca-miR-411-5p*	MIMAT0003329	uaguagaccguauagcguacg	cagtagtagaccgtatagcgt
*eca-miR-412-3p*	MIMAT0013155	uucaccugguccacuagccg	gcagttcacctggtcca
*eca-miR-412-5p*	MIMAT0026557	uggucgaccaguuggaaaguaau	cagtggtcgaccagttg
*eca-miR-432-5p*	MIMAT0013157	ucuuggaguaggucauugggugg	cagtcttggagtaggtcattg
*eca-miR-433-3p*	MIMAT0013158	aucaugaugggcuccucggugu	catgatgggctcctcg
*eca-miR-485-3p*	MIMAT0013160	gucauacacggcucuccucucu	gcaggtcatacacggct
*eca-miR-485-5p*	MIMAT0013159	agaggcuggccgugaugaauuc	ggctggccgtgatga
*eca-miR-487a-5p*	MIMAT0026559	gugguuaucccugcuguguucg	caggtggttatccctgct
*eca-miR-487b-3p*	MIMAT0013162	aaucguacagggucauccacuu	cagaatcgtacagggtcatc
*eca-miR-493b-5p*	MIMAT0002813	uuguacaugguaggcuuucauu	gcgcagttgtacatggtag
*eca-miR-543-3p*	MIMAT0013169	aaacauucgcggugcacuucuu	gcagaaacattcgcggtg
*hsa-miR-1247-3p*	MIMAT0022721	ccccgggaacgucgagacuggagc	cgggaacgtcgagac
*hsa-miR-154-5p*	MIMAT0000452	uagguuauccguguugccuucg	gcagtaggttatccgtgttg

* mirbase.org; Release 21. Primers were designed using miRprimer software (version 2.0).
